# Ten steps to get started in Genome Assembly and Annotation

**DOI:** 10.12688/f1000research.13598.1

**Published:** 2018-02-05

**Authors:** Victoria Dominguez Del Angel, Erik Hjerde, Lieven Sterck, Salvadors Capella-Gutierrez, Cederic Notredame, Olga Vinnere Pettersson, Joelle Amselem, Laurent Bouri, Stephanie Bocs, Christophe Klopp, Jean-Francois Gibrat, Anna Vlasova, Brane L. Leskosek, Lucile Soler, Mahesh Binzer-Panchal, Henrik Lantz

**Affiliations:** 1 Institut Français de Bioinformatique, UMS3601-CNRS, Université Paris-Saclay, Orsay, 91403, France; 2 Department of Chemistry, Norstruct, UiT The Arctic University of Norway, Tromsø, 9019, Norway; 3 Department of Plant Biotechnology and Bioinformatics, Ghent University, Technologiepark 927, 9052 Ghent, Belgium; 4 VIB-UGent Center for Plant Systems Biology, Ghent University - VIB, Technologiepark 927, 9052 Ghent, Belgium; 5 Spanish National Bioinformatics Institute (INB), Barcelona, Spain; 6 Barcelona Supercomputing Center (BSC), Centro Nacional de Supercomputación, Barcelona, Spain; 7 Centre for Genomic Regulation (CRG), The Barcelona Institute for Science and Technology , Barcelona, Spain; 8 Universitat Pompeu Fabra (UPF), Barcelona, Spain; 9 Uppsala Genome Center, NGI/SciLifeLab, Department of Immunology, Genetics and Pathology, Uppsala University, Uppsala, SE-752 37 , Sweden; 10 URGI, INRA, Université Paris-Saclay, Versailles, 78026, France; 11 CIRAD, UMR AGAP, Montpellier, 34398, France; 12 AGAP, Cirad, INRA, Montpellier SupAgro, Universite Montpellier, Montpellier, France; 13 South Green Bioinformatics Platform, Montpellier, France; 14 Genotoul Bioinfo, MIAT, INRA Toulouse, Castanet-Tolosan, France; 15 Unité de recherche , INRA, Université Paris-Saclay, 78350 Jouy-en-Josas, France; 16 Faculty of Medicine, Institute for Biostatistics and Medical Informatics, University of Ljubljana, Ljubljana, Slovenia; 17 IMBIM/NBIS/SciLifeLab, Uppsala University, Uppsala, Sweden

**Keywords:** Genome, Assembly, Annotation, FAIR, NGS, Workflows, DNA

## Abstract

As a part of the ELIXIR-EXCELERATE efforts in capacity building, we present here 10 steps to facilitate researchers getting started in genome assembly and genome annotation. The guidelines given are broadly applicable, intended to be stable over time, and cover all aspects from start to finish of a general assembly and annotation project.

Intrinsic properties of genomes are discussed, as is the importance of using high quality DNA. Different sequencing technologies and generally applicable workflows for genome assembly are also detailed. We cover structural and functional annotation and encourage readers to also annotate transposable elements, something that is often omitted from annotation workflows. The importance of data management is stressed, and we give advice on where to submit data and how to make your results Findable, Accessible, Interoperable, and Reusable (FAIR).

## Introduction

The advice here presented is based on a need seen while working in the ELIXIR-EXCELERATE task “Capacity Building in Genome Assembly and Annotation”. In this capacity we have held courses and workshops in several European countries and have encountered many users in need of a document to support them when they plan and execute their projects. With these 10 steps we aim to fill this need.

In a *de novo* genome assembly and annotation project, the nucleotide sequence of a genome is first assembled, as completely as possible, and then annotated. The annotation process infers the structure and function of the assembled sequences. Protein-coding genes are often annotated first, but other features, such as non-coding RNAs or presence of regulatory or repetitive sequences, can also be annotated.

With the advances in sequencing technologies it has become much more feasible, and affordable, to assemble and annotate the genomic sequence of most organisms, including large eukaryote genomes ^1, 2^. However, high quality genome assembly and annotation still represent a major challenge. Considerable time and computational resources are often needed, and researchers have to be prepared to provide these resources in order to be successful. Assembly and annotation of small genomes e.g., bacterias and fungi, can often be performed with fairly small resources and a limited time commitment, but eukaryotic genome projects often take months or even years to finish, especially when no reference genomes can be used for these tasks. The mere running of assembly or annotation tools can take several weeks (see Section 3 for examples).

Considering the amount of time, knowledge, and resources required by these projects, an early question you need to ask yourself is: “Do I really need an assembled and annotated genome?” In many cases an assembled transcriptome, or perhaps a re-sequencing approach based on the genomic sequence of a related species, can be enough to answer your scientific questions. These two approaches both constitute solutions requiring much less resources, both in amount of sequencing data needed and in regards to compute hours, but are more limited and do not offer as many possibilities as an annotated genome does. In the event that a genome draft has a significant added value to address the problem, one should consider whether sufficient financial and computational resources are available to produce a genome of satisfactory quality.

For those that indeed have decided to embark upon a genome assembly and/or annotation project, we provide, here, a set of good practices intended to facilitate the project completion. The target audience is someone entering this field for the first time, and we strive to answer his/her beginner questions. We split the information up into different sections for the reader to easily find the parts that are of their particular interest. The guidelines are meant to be broadly applicable to multiple software pipelines and sequencing technologies and do not focus on specifics, as the field is rapidly changing and discussion on current tools could quickly become outdated.

A checklist of things to keep in mind when starting a genome project:

- For the DNA extraction, select an individual which is a good representative of the species, and able to provide enough DNA.
- Extract more DNA than you think you need, or save tissue to use for DNA extraction later. If you need to produce more data later, it is critical to be able to use the same DNA to make sure the data assembles together.
- Remember to extract RNA and order RNA-sequencing if you want to use assembled transcripts in your annotation (which is strongly recommended). If possible, extract RNA from the same individual as used in the DNA extraction to make sure that the RNA-seq reads will map well to your assembly.
- Decide early on which sequencing technology you will be using, and also consider which assembly tools you want to try. These two choices will greatly influence what kind of compute resources you will need, and you do not want to end in a situation where you have data that you cannot analyze anywhere. Plan compute resources accordingly.

## 1. Investigate the properties of the genome you study

Every assembly or annotation project is different. Distinctive properties of the genome are the main reason behind this. To get an idea of the complexity of an assembly or annotation project, it is worth looking into these properties before starting. Here, we will discuss some genome properties, and how they influence the type and amount of data needed, as well as the complexity of analyses.

### Genome size

To assemble a genome, a certain amount of sequences (also called reads) is needed. For example, for Illumina sequencing (see Illumina Genome Assembly below), a number of >60x sequence depth is often mentioned. This means that the number of total nucleotides in the reads need to be at least 60 times the number of nucleotides in the genome. From this it follows that the bigger the genome, the more data is needed. You need to get an estimate of the genome size before ordering sequence data, perhaps from flow cytometry studies, or if no better data exists, by investigating what is the genome size of closely related and already assembled species. This is an important value to bring to the sequencing facility, as the genome size will greatly influence the amount of data that needs to be ordered. Available databases for approximate genome sizes are available for plants ( http://data.kew.org/cvalues), for fungi ( http://www.zbi.ee/fungal-genomesize), and for animals ( http://www.genomesize.com).

### Repeats

Repeats are regions of the genome that occur in multiple copies, potentially at different locations in the genome. Amount and distribution of repeats in a genome hugely influences the genome assembly results, simply because reads from these different repeats are very similar, and the assembly tools cannot distinguish between them. This can lead to mis-assemblies, where regions that are distant in the genome are assembled together, or an incorrect estimate of the size or number of copies of the repeats themselves ^3^. Very often a high repeat content leads to a fragmented assembly, as the assembly tools cannot determine the correct assembly of these regions and simply stop extending the contigs at the border of the repeats ^4^. To resolve the assembly of repeats, reads need to be long enough to also include the unique sequences flanking the repeats. It can therefore be a good idea to order data from a long-read technology, if you know that you are working with a genome with a high content in repeats.

### Heterozygosity

Assembly programs in general try to collapse allelic differences into one consensus sequence, so that the final assembly that is reported is haploid. If the genome is highly heterozygous, sequence reads from homologous alleles can be too different to be assembled together and these alleles will then be assembled separately. This means that heterozygous regions might be reported twice for diploid organisms, while less variable regions will only be reported once, or that the assembly simply fails at these variable regions ^5^. Highly heterozygous genomes can lead to more fragmented assemblies, or create doubt about the homology of the contigs. Large population sizes tend to lead to high heterozygosity levels. For instance, marine organisms often have high heterozygosity levels and are often problematic to assemble. It is recommended to sequence inbred individuals, if possible.  

### Ploidy level

If possible, it is better to sequence haploid tissues (true for bacteria and many fungi) since, this will essentially remove problems caused by heterozygosity. Diploid tissues, which will be the case for most animals and plants, is fine and usually manageable, while tetraploidy and above has the potential to greatly increase the number of present alleles, which likely will result in a more fragmented assembly (see heterozygosity above). Diploid-aware assemblers using long reads can help, but keep in mind that correct assembly of diploid genomes might require higher coverage.  

### GC-content

Extremely low or extremely high GC-content in a genomic region is known to cause a problem for Illumina sequencing, resulting in low or no coverage in those regions ^6^. This can be compensated by an increased coverage, or the use of a sequencing technology that does not exhibit that bias (i.e., PacBio or Nanopore). If you are working with an organism with a known low or high GC-content, we would recommend using a sequencing technology that does not exhibit any bias in this regard.

## 2. Extract high quality DNA

Intrinsic properties of the genome are not the only consideration before sequencing. There are also other aspects that need careful planning. The extraction of high quality DNA is one such aspect that is of utmost importance. We discuss DNA extraction in some detail below, but also end this section with a short list of other pre-assembly considerations important to keep in mind when starting an assembly project.

### DNA quality requirements for *de novo* sequencing

Few researchers are aware of the fact that to get a good reference genome one must start with good quality material. It must be immediately pointed out that PCR-quality DNA and NGS-quality DNA are two completely different things ^7^.

In general, we recommend using long-read technologies (see also Section 3 below) when carrying out genome assembly. For these technologies, it is crucial to use best quality High Molecular Weight (HMW) DNA, which is obtained mainly from fresh material. The lack of a good starting material will limit the choice of sequencing technology and will affect the quality of obtained data.

The most important DNA quality parameters for NGS are chemical purity and structural integrity of the sample.

### Chemical purity

DNA extracts often contain carry-over contaminants originating either from the starting material or from the DNA extraction procedure itself. Examples of sample-related contaminants are polysaccharides, proteoglycans, proteins, secondary metabolites, polyphenols, humic acids, pigments, etc. For instance, fungal, plant and bacterial samples can contain high levels of polysaccharides, plants are notorious for their polyphenols, and insect samples are usually contaminated by polysaccharides, proteins and pigments, and so on. All these contaminants can impair the efficacy of library preparation in any technology, but this is especially true for Illumina Mate Pair libraries and PCR-free libraries (both PacBio and ONT). For conventional short-read technology sequencing where a PCR step is involved in the library prep, this hurdle is partly overcome by the amplification step during the library construction. However, it can happen that the library complexity of a contaminated sample can be reduced due to lower efficacy of the reaction. It is widely known in the PacBio community that samples rich in contaminants can fail or underperform in the sequencing process, since there is no PCR step in the library preparation and sequencing workflow.

The way to address the contamination issue is to use an appropriate DNA extraction protocol taking into account the expected type of contaminants present in the sample (native contaminants). CTAB (cetyl trimethylammonium bromide) extraction is highly recommended for DNA extraction from fungi, mollusks and plants; at a certain salt concentration CTAB helps to differentially extract DNA from solutions containing high level of polysaccharides ^8^ . For protein rich tissues, adding beta-mercaptoethanol (disrupting disulphide bonds in protein molecules) and optimization of Proteinase K treatment is recommended ^9^. For plants, it is important to always use a combination of beta-mercaptoethanol (to prevent polyphenols from oxidizing and binding to DNA) and PVPP (polyvinyl polypyrrolidone; to absorb polyphenols and other aromatic compounds) ^10^. For animal and human samples, it is advised to use tissues with low fat and connective tissue content.

### Structural integrity of DNA

Aside from native contaminants, phenol, ethanol and salts can be introduced during the DNA extraction procedure. Incomplete removal of phenol, or not using fresh phenol will harm DNA (e.g. introducing nicks making the nucleic acid more fragile); it can also impair enzymes used in downstream procedures, as can incompletely removed ethanol. High salt concentrations (e.g. EDTA carry-over) can potentially lower efficacy of any downstream enzymatic reactions.

A second important issue is the DNA structural integrity, which is especially important for long-read sequencing technologies. DNA can become fragile due to nicking introduced during DNA extraction, or using storage buffer with inappropriate pH. Prolonged DNA storage in water and above -20°C is not recommended; it increases the DNA degradation risk due to hydrolysis. High molecular weight DNA is fragile; therefore using gentle handling (vortexing at minimal speed, pipetting with wide-bore pipette tips, transportation in a solid frozen stage) is advised. It is also advisable to keep the number of freeze-thaw cycles to a minimum, since ice-crystals can mechanically damage the DNA. For the same reason, one should avoid DNA extraction protocols involving harsh bead-beating treatment during tissue homogenization.

It must be also pointed out that RNA contamination of DNA samples must be avoided. Most NGS DNA library preps can only efficiently utilize double-stranded DNA. Having RNA contamination in the sample will overestimate the library nucleic acid molecules concentration. That is especially true for PacBio and 10X Chromium libraries.

To summarize, it is always worth investing time in getting a high quality DNA prep – it can potentially save lots of time and money that would otherwise be spent on sequencing troubleshooting, ordering more data, or, if ordering more data is not possible, trying to assemble a genome with a coverage that is lower than expected.

### Other considerations

- Pooling of individuals – For some organisms it can be difficult to extract a sufficient amount of DNA, and in these cases it might be tempting to pool several individuals before extraction. Note that this will increase the genetic variability of the extraction, and can lead to a more fragmented assembly, just like high levels of heterozygosity would. In general pooling should be avoided, but if it is done, using closely related and/or inbred individuals is recommended.
- Whole Genome Amplification (WGA) – In cases where perhaps only a few cells are available, the genomic DNA needs to be amplified to be sequenced. This will often result in uneven coverage, and in the case of amplification methods relying on multiple strand displacement, artificial so called chimeric sequences consisting of fused unrelated sequences can be created ^11^. Be aware that this can cause mis-assemblies. If possible, use an assembly tool designed to work with amplified DNA, for example SPAdes ^12^.
- Presence of other organisms – Contamination is always a risk when working with DNA. For genome assembly, contamination can be introduced in the lab at the DNA extraction stage, or other organisms can be present in the tissue used, e.g. contaminants and/or symbionts. Care should be taken to make sure that the DNA of other organisms does not occur in higher concentrations than the DNA of interest, as many reads will then be from the contaminant rather than the genome of the studied organism. Small amounts of contamination are rarely a problem as these reads can be filtered out at the read quality control step or after assembly, unless the contaminants are highly similar to the studied organism.
- Organelle DNA - Some tissues are so rich in mitochondria or chloroplasts that the organelle DNA occurs in higher concentrations than the nuclear DNA. This can lead to lower coverage of the nuclear genome in your sequences. If you have a choice, choose a tissue with a higher ratio of nuclear over organelle DNA.

## 3. Choose an appropriate sequencing technology

**Figure 1.**
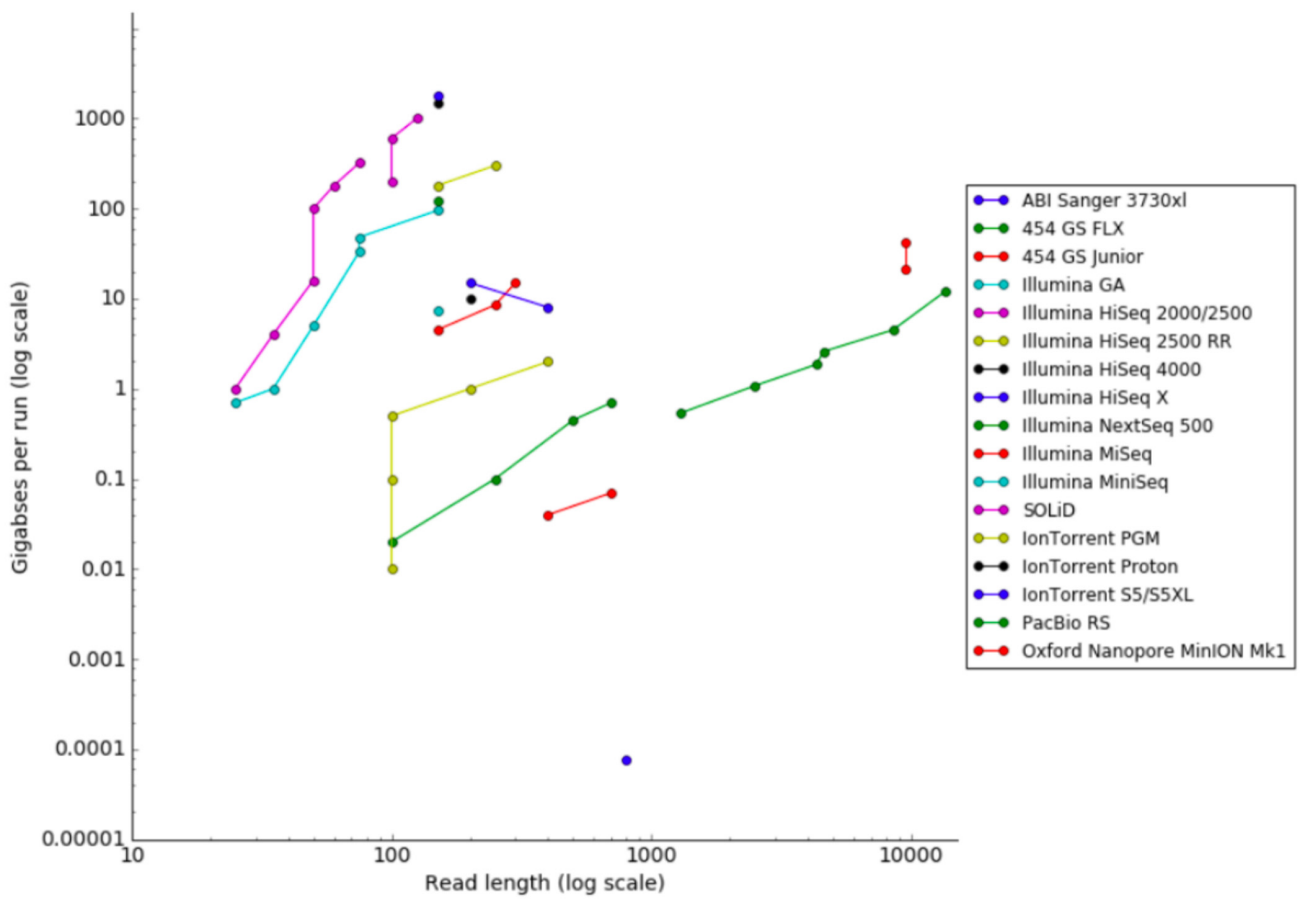
Timeline and comparison of different sequencing technologies. The data is based on the throughput metrics for the different platforms since their first instrument version came out. The figure visualises the results by plotting throughput in raw bases versus read length. Data released under CC BY 4.0 International license. doi 10.6084/m9.figshare.100940.

The choice of which sequencing technology to use is an important one ( Figure 1). It will influence the cost and success of the assembly process to a large degree. In this section, we will discuss the currently available and most commonly used options, and also some supporting technologies. It is worth mentioning that assembly programs are often very specific in what type of data they accept, and might not be able to analyze reads from different sequencing technologies together. You should decide how to analyze your sequence data before you order it, to decrease the risk of needing to order, and wait for, more DNA/RNA material just to be able to perform your analyses.

### First generation sequencing (FGS)

These technologies started with the Sanger sequencing method developed by Frederick Sanger and colleagues in 1977. The method is based on selective incorporation of chain-terminating dideoxynucleotides by DNA polymerase during *in vitro* DNA replication. FGS technologies were the most widely used for approximately 30 years ^13, 14^.

During the last decade, the Sanger method has been replaced by High-Throughput Sequencing platforms (HTS), in particular by Second-Generation Sequencing (SGS), which is much less expensive. However, the Sanger method remains widely used in smaller-scale projects and for closing gaps between contigs generated by HTS platforms.

### SGS and Third-Generation Sequencing

The SGS have dominated the market, thanks to their ability to produce enormous volumes of data cheaply. Examples are the Illumina or Ion Torrent sequencers. Many remarkable projects like the 1000 Genomes Project ^15^ and the Human Microbiome Project ^16^ have been finished thanks to SGS technologies. However, some genes and important regions of interest are often not assembled correctly, mainly due to the presences of repeat elements in the sequences ^17^. A promising solution is Third-Generation-Sequencing (TGS) based on long reads ^18^. TGS technologies have been used for the reconstruction of highly contiguous regions in eukaryotic genomes ^19, 20^ and *de novo* microbial genomes with high precision ^21^. In terms of resequencing, the TSG technology has generated detailed maps of the structural variations in multiple species and has covered many of the gaps in the human reference genome ^22, 23^.

Currently, the two most important third-generation DNA sequencing technologies are Pacific Biosciences (PacBio) Single Molecule Real Time (SMRT) and Oxford Nanopore Technology (ONT) ^24^. These technologies can produce long reads averaging between 10,000 to 15,000bp, with some reads exceeding 100,000bp.

However, these long reads exhibit per sequence error rates up to 10% to 15%, requiring a preliminary stage of correction before **or after** the assembly process. In fact, long read assembly has caused a paradigm shift in whole-genome assembly in terms of algorithms, software pipelines and supporting steps ^25^.

### Supporting technologies

There are also supporting technologies, most of which are used to improve the contiguity of already existing genome assemblies. These include optical mapping methods (e.g., BioNano), linked-read technologies (e.g., 10X Genomics Chromium system), or the genome folding-based approach of HiC ^26^. In a rapidly changing field, it is difficult to recommend one of these technologies over the others. We advise researchers interested in assembling large genomes to read up on the current status of these methods when ordering sequence data, and remember to budget for them. For researchers interested in large-scale structural changes, the improvements of contiguity provided by these methods will be of extra interest.

Long reads definitely have an advantage over shorter reads when used in genome assembly as they deal with repeats much better. In practice, this often leads to less fragmented assemblies, which is what most researchers are aiming for. The problems with third generation technologies are a higher price, a lack of availability in some countries, and sometimes higher requirements in terms of DNA amount and quality. Unless these complicating factors prevents the use of third generation long read technologies in your research project, we strongly recommend them over short read technologies. That being said, a combination of both might be even better, as the shorter reads have a different error profile and can be used to correct the longer ones ^27^ (see Section 5).

## 4. Estimate the necessary computational resources

To succeed in a genome assembly and annotation project you need to have sufficient compute resources. The resource demands are different between assembly and annotation, and different tools also have very different requirements, but some generalities can be observed (for examples, see Table 1).

**Table 1.** Examples of time and computer resources used by software dedicated to assembly and annotation. SPAdes is an assembler designed for the assembly of small genomes using short reads. Smartdenovo is a *de novo* assembler for PacBio and Oxford Nanopore (ONT) data. The REPET package is a software suite dedicated to detect, classify and annotate repeats. EuGene is an open integrative gene finder for eukaryotic and prokaryotic genomes. Processing time and RAM used will be affected by amount of input data, complexity of data, and genome size.

Reference Genome	Size	Software	Input (space used on disk)	CPU/RAM Available	Real time	Max RAM Used
***Aliivibrio wodanis***	4 972 754 bp	SPAdes v3.10	200x Illumina reads (760 MB)	4 CPU/16GB RAM	2h17m3s	2,94GB
				12 CPU/256GB RAM	38m8s	9,37GB
***Caenorhabditis*** ***elegans***	100 272 607 bp	Smartdenovo	20x Pacbio P6C4 Corrected long reads (1,9 GB)	8 CPU/16GB RAM	24m47s	1,92GB
			80x Pacbio P6C4 Corrected long reads (7,6 GB)	8 CPU/16GB RAM	5h38m16s	7,29GB
		REPET v2.5	*C. Elegans* genome (100 MB) Repbase aa 20.05 (20 MB) Pfam 27 (GypsyDB) (1,2 GB) rRNA from eukaryota (2,6 MB)	8 CPU/16 GB RAM	1h53m11s + 19h9m40s	8,96GB
		Eugene v4.2a	*C. Elegans* genome (100 MB) Repbase aa 20.05 (20 MB) Proteins sequences (swissprot) (2,8 MB) ESTs sequences (29 MB)	8 CPU/32 GB RAM	5h2m30s	16,94GB
***Arabidopsis thaliana***	134 634 692 bp	Smartdenovo	20x Pacbio P5C3 corrected long reads (2,7 GB)	8 CPU/16GB RAM	1h16m20s	2,4GB
		REPET v2.5	*A. Thaliana* genome (130 MB) Repbase aa 20.05 (20 MB) Pfam 27 (GypsyDB) (1,2 GB) rRNA from eukaryota (2,6 MB)	8 CPU/16 GB RAM	5h6m23s + 33h10m34s	10,25GB
		Eugene v4.2a	*A. Thaliana* genome (130 MB) Repbase aa 20.05 (20 MB) Proteins sequences (swissprot) (9,2 MB) ESTs sequences (31 MB)	8 CPU/32 GB RAM	6h17m18s	17,25GB
***Theobroma*** ***cacao***	324 761 211 bp	Eugene v4.2a	*T. Cacao* genome (315 MB) Repbase aa 20.05 (20 MB) Proteins sequences (swissprot) (31 MB) ESTs sequences (103 MB)	8 CPU/188 GB RAM	41h27m13s	72,5GB

For genome assembly, running times and memory requirements will increase with the amount of data. As more data is needed for large genomes, there is thus also a correlation between genome size and running time/memory requirements. Only a small subset of available assembly programs can distribute the assembly into several processes and run them in parallel on several compute nodes. Tools that cannot do this tend to require a lot of memory on a single node, while programs that can split the process need less memory in each individual node, but do on the other work most efficiently when several nodes are available. It is therefore important to select the proper assembly tools early in a project, and make sure that there are enough available compute resources of the right type to run these tools.

Annotation has a different profile when it comes to computer resource use compared to assembly. When external data such as RNA-seq or protein sequences are used (something that is strongly recommended), mapping these sequences to the genome is a major part of the annotation process. Mapping is computationally intense, and it is highly preferable to use annotation tools that can run on several nodes in parallel.

Regarding storage, usually no extra consideration needs to be taken for assembly or annotation projects compared to other NGS projects. Intermediate files are often much larger than the final results, but can often be safely deleted once the run is finished.

## 5. Assemble your genome

**Figure 2.**
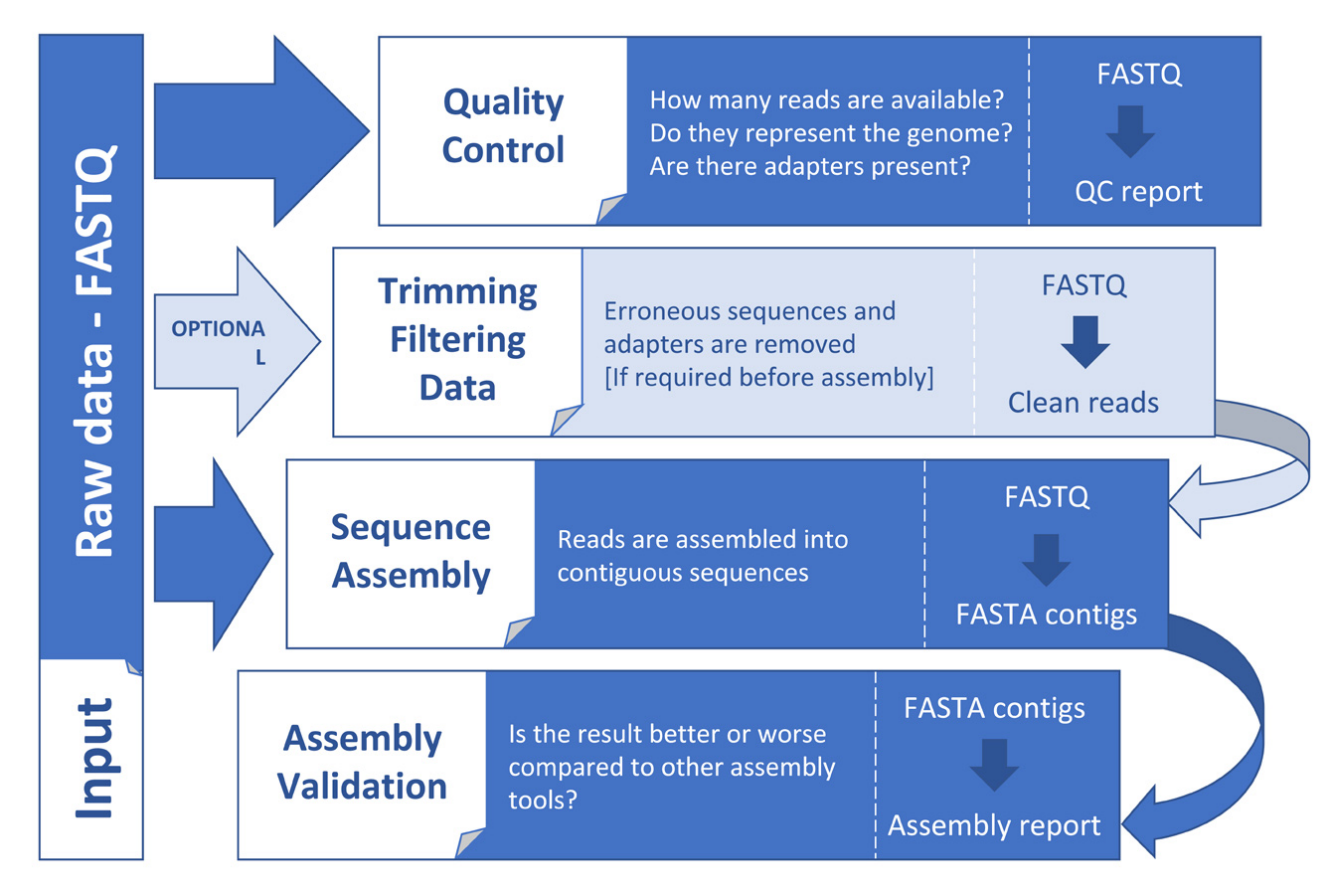
General steps in a genome assembly workflow. Input and output data are indicated for each step.

In general, irrespective of the sequencing technology you choose, you would follow the same workflow ( Figure 2). In the quality control (QC) stage the sequence reads are examined for overall quality and presence of adapters. Presence of contaminants can also be examined. In the assembly stage, several assemblers are often tried in parallel and the results are then compared in the assembly validation step, where mis-assemblies also can be identified and corrected. Often, assemblers are rerun with new parameters based on the results of the assembly validation. The aim is usually to create a genome assembly with the longest possible assembled sequences (least fragmented assembly) with the smallest number of mis-assemblies.

Quality control of reads and the actual genome assembly are different for the Illumina technology compared with long read technologies. These technologies will be discussed separately hereafter. We end this section with a discussion about assembly validation, which is similar for all technologies.

### Illumina Genome assembly

The most common approach to perform genome assemblies is *de novo* assembly, where the genome is reconstructed exclusively from the information of overlapping reads. For prokaryotes, it is also common to assemble with a reference genome, e.g., when complete strain collections are sequenced. The reference sequence can either be used as a template to 1) guide the mapping of reads, or 2) reorder the *de novo* assembled contigs.

In general, Illumina sequencing technology produces large amounts of high quality short sequence reads. The adapter and multiplex index sequences are screened for and removed after the base calling on the sequencing machine. However, it is highly recommended to assess the raw sequence data quality prior to assembly. Poor quality reads, ambiguous base calling, contamination, biases in the data and even technical issues on the sequencing chip, are some, but not all, possible technical errors that can be detected early and corrected ^28^. Also, if the sequencing libraries contain very short fragments, it is likely that the sequencing reaction will continue past the DNA insert and into the adapter in the 3' end, a process known as adapter read-through, which may escape the adapter screening step on the sequencing machine ^29^.

### Assessing the quality of Illumina short reads

Assessing the quality of the sequence data is important, as it may affect downstream applications and potentially lead to erroneous conclusions. Base calling accuracy measures the probability that a given base is called incorrectly, and is commonly measured by the Phred quality score (Q score). Several tools are available for the quality assessment. FastQC ^30^ is a commonly used tool that can be run both from the command line or through an interactive graphical user interface (GUI). It produces plots and statistics showing, among others, the average and range of the sequence quality values across the reads, over-represented sequences and k-mers which in total can help the user interpret data quality. k-mers represent all subsequences of length k in a sequence read. Most methods for assembling or mapping reads are based on the use of k-mers. More in depth analysis of k-mers can also be performed, for example using KAT ^31^ to identify error levels, biases and contamination, and this also comes highly recommended.

### Pre-processing of raw data

After having investigated the sequence data quality, informed decisions on downstream operations can be made. We would in general recommend that adapters are removed, although there are also assemblers that prefer working with the raw data, including potential adapter sequences. It is highly recommended that the user studies the assembler documentation to determine whether the program requires quality-trimmed data or not. If trimming is required by the assembler, it would be sensible to omit poor quality data from further analysis by trimming low quality read ends and filtering of low quality reads. A variety of tools are available, such as PRINSEQ ^32^, which offers a standalone command-line version, a version with a GUI and an online web based service, and Trimmomatic ^33^.

Illumina machines produce a wide range of read numbers, from 10 millions up to 20 billions (NovaSeq). Reducing the sequence coverage by subsampling for deeply sequenced genomes is recommended, as *de Brujin* assemblers work best around 60-80x coverage ^34^. High coverage in a particular genome location will increase the probability that this location is seen as a sequencing error or sequencing errors can propagate and start to look like true sequence. BBnorm ^35^, a member of the BBTools package, is a common kmer-based normalisation tool that can normalise highly covered regions to the expected coverage.

### Short reads genome assembly

For the *de novo* assembly of short reads, the most commonly used algorithms are based on *de Bruijn* graphs, although other algorithms such as Overlap Layout Consensus (OLC) ^36^ are still being used. One of the advantages of *de Bruijn* graph over OLC is that it consumes less computational time and memory. Depending on the complexity of the genome to be assembled such as size, repeat-content, polyploidy, a proper tool should be selected. Some assembly tools, such as SPAdes ^12^, work best with smaller amounts of data and are thus well adapted for bacterial projects, while others handle large amounts of data well and can be used for any type of project. These include allpaths-LG ^37^ and Masurca ^38^. Note that with large amounts of data, available RAM will be a limiting factor.

The characteristics of the genomes being assembled have a greater impact on the results than the choice of the algorithm. Haploid genomes with no sequence repeats will be much easier to reconstruct than genomes of polyploids or genomes with many sequence repeats e.g. many plants species. The GAGE-B study ^39^ showed that assembly software performing well on one organism, performed poorly on another organism. Hence, it is wise to test several approaches; different software, assembly with or without pre-processing of the sequence data, and also with different parameter settings. Another approach that will have impact on the assembly is the use of mate pair sequencing. This enables the generation of long-insert paired-end DNA libraries with fragments up to 15 kb, and can be particularly useful in *de novo* sequencing. The large inserts can span across regions problematic to the assembler such as repetitive elements, and anchor the paired reads in unique parts of the DNA, and reduce the number of contigs and scaffolds. Despite the enormous development in this field, it is still challenging to assemble large genomes from short reads. Further improvements, both in the assembly technology, but also in increasing read length and in fragment size is needed for more accurate reconstruction of genomes.

### Long read genome assembly

TGS developed by Pacific Biosciences or Oxford Nanopore is able to produce long reads with average fragment lengths of over 10,000 base-pairs that can be advantageously used to improve the genome assembly ^40^. In fact, long reads can span stretches of repetitive regions and thus produce a more contiguous reconstruction of the genome. However, raw long reads have a high rate of sequencing error (5–20%). As a result, some long read assemblers opt to correct these errors prior to assembly.

There are two main families of assemblers based on long reads:

- Long Reads Only assembler (LRO)
- Short and Long Reads combined assembler (SLR)

In general, LRO assemblers are based on the OLC algorithm. First, this algorithm produces alignments between long reads. Then it calculates the best overlap graph, and finally it generates the consensus sequence of the contigs from the graph. LRO assemblers require more sequencing coverage (minimum ~50X) from the long reads dataset than SLR assemblers. Schematically, SLR assemblers instead generate a *de Bruijn* graph pre-assembly using short reads, then the long reads are used to improve the pre-assembly by closing gaps, ordering contigs, and resolving repetitive regions. It is worth noting that some long reads assemblers require corrected long reads as input. Software to correct long reads are based on two strategies. The first strategy consists of aligning long reads against themselves. The second one uses short reads to correct long reads.

A document with guideline practices for long-reads genome assemblies is available ^41^. This document shows the performance of long read assembly benchmarked against 4 reference genomes: *Acinetobacter DP1*, *Escherichia coli* K12 MG1655, *Saccharomyces cerevisae* W303 and *Caenorhabditis elegans* (sequenced in different TGS platforms and under different conditions). Among the 11 tools that have been evaluated, 8 use only long reads as input data, while the 3 others can assemble genome using a mix of long and short reads. The tests show that it is strongly recommended to use a long read correction software before the assembly ^42^.

### Assembly polishing

Although an error correction step may have been part of the assembler pipeline, errors can still be present in the assembly, particularly in long read assemblies. Polishing draft assemblies with either short or long reads can help to improve local base accuracy in particular correcting base calls and small insertion-deletion errors, and also resolve some mis-assemblies caused by poor reads alignment during the assembly ^43^.

### Scaffolding and gap filling

In scaffolding, assembled contigs are stitched together based on information from paired short reads. The unknown sequence between the contigs will be filled with Ns. If matching reads are instead used to join contigs together, for example long reads, actual sequence will fill in the gaps, and this is referred to as gap filling. In the case of an existing scaffolded assembly, long reads can also be used to replace the N-regions. Note that misassemblies in an existing assembly need to be broken prior to scaffolding in order to join the correct contigs together. Scaffolding and gap filling can be performed with low coverage ^44^.

### Determining whether the assembly is ready for annotation

Determining if the assembly is ready for annotation is a key step towards successful genome annotation. Errors in assemblies occur for many reasons. Genomic regions can be incorrectly discarded as being fallacies or repeats. Others can be spliced together in the wrong places or in the wrong orientation. Unfortunately, there are few ways to distinguish what is real, what is missing, and what is an experimental artefact. There are, however, some statistics that often are used when choosing between assemblies, and some ways of identifying and removing potential problems.

N50 is often used as a standard metric to evaluate an assembly ^45^. N50 is the length of the smallest contig, after they have been ranked from longest to smallest, such that the sum of contig lengths up to it covers 50% of the total size of all contigs. It is thus a measure of contiguity, with higher numbers indicating lower levels of fragmentation. It is important to note that N50 is not a measure of correctness. So-called aggressive assemblers may produce longer contigs and scaffolds than conservative assemblers, but are also more likely to join regions in the wrong order and orientation. We recommend to compare the output from different assemblers (and of trimmed/filtered data). Assembly evaluation tools, such as Quast ^46^, compare the metrics between assemblies, and allow the user to make educated choices to further improve and select the best assembly. If a reference sequence is available, Quast can also describe mis-assemblies and structural variations relative to the reference. If paired Illumina data is available, tools such as Reapr ^47^ or FRCBam ^48^ can be used to evaluate assemblies and to identify which assembly has the least amount of misassemblies. If other organisms were present in the reads (contaminants or symbionts) and have been assembled together with the other reads, these contigs can be identified using for example Blobtools ^49^ and removed, if necessary. To determine how many protein coding genes have been assembled, BUSCO ^50^ is very useful. This tool looks for genes that should be present in a genome of the investigated taxonomic lineage type, and reports the number of complete and fragmented genes found. Choosing the assembly with the highest percentage of complete genes could be given greater importance if the purpose of the genome project is to investigate protein coding genes.

Knowing when to stop assembly and moving into annotation is one of the most difficult decisions to take in genome assembly projects. It is always possible to try one more tool or one more setting, and this wish of wanting to improve the assembly just a little bit more can delay these types of projects substantially. It is best to have a goal in mind before starting assembly, and to stop when that goal has been reached. If you feel that you can answer the questions you had before starting, then the assembly is good enough for your purposes and it is probably time to move into annotation. It is always possible to release a new and improved version of the genome later. Be aware that any changes to a genome assembly will most likely necessitate annotation to be re-started from scratch, and you should therefore be sure to “freeze” the assembly completely before starting annotation.

## 6. Do not neglect to annotate Transposable Elements

The genome annotation stage starts with repeat identification and masking.

There are two different types of repeat sequences: ‘low-complexity’ sequences (such as homopolymeric runs of nucleotides) and **transposable elements**. Transposable Elements (TEs) are key contributors to genome structure of almost all eukaryotic genomes (animals, plants, fungi). Their abundance, up to 90% of some genomes such as wheat ^51^, is usually correlated with genome size and organization. TEs ability to move and to accumulate in genomes, make them a major players of genome structure, plasticity, genetic variations and evolution. Interestingly, they can affect gene expression, structure and function when their insertion occurs in the vicinity of genes ^52^ and sometimes through epigenetic mechanisms ^53^.

TEs are classified in two classes including subclasses, orders and superfamilies according to mechanistic and enzymatic criteria. These two classes are based on their mechanism of transposition using a copy-and-paste (Class I) or cut-and-paste mechanisms (Class II) through RNA or DNA intermediates respectively ^54^.

TE annotation is nowadays considered as a major task in genome projects and should be undertaken before any other genome annotation task such as gene prediction. Consequently, there has been a growing interest in developing new methods allowing an efficient computational detection, annotation, and analysis of these TEs, in particular when they are nested and degenerated. Many software have been developed to detect and annotate TEs ^55^. One of the best known is RepeatMasker, which harnesses nhmmer, cross_match, ABBlast/WUBlast, RMBlast and Decypher as search engines and uses curated libraries of repeats, currently supporting Dfam (profile HMM library) and Repbase ^56, 57^.

Another important tool is the REPET package, one of the most used tools for large eukaryotic genomes with more than 50 genomes analyzed in the framework of international consortia. The REPET package is a suite of pipelines and tools designed to tackle biological issues at the genomic scale.

REPET consists of two main pipelines: TEdenovo and TEannot. First, TEdenovo efficiently detects classified TEs (TEdenovo pipeline), then TEannot annotates TEs, including nested and degenerated copies ^58^.

Depending on the complexity and number of detected TEs, it might be possible that additional rounds of TEs identification and removal are needed once the initial gene set has been produced. It is a common practice to analyze the functional annotation of the initial gene set to detect those genes which are primarily annotated with terms associated to TEs activity. Those genes can be safely removed if they do not have homologous sequences in relative species and/or their homologous sequences have been annotated as TEs related ^59^.

## 7. Annotate genes with high quality experimental evidence

### 7.1. Structural annotation – where are the genes and what do they look like?

A raw genomic sequence is to most biologists of no great value as such. Genome annotation consists of attaching biological meaningful information to genome sequences by analyzing their sequence structure and composition as well as to consider what we know from closely related species, which can be used as reference. While genome annotation involves characterizing a plethora of biologically significant elements in a genomic sequence, most of the attention is spent on the correct identification of protein coding genes. This is not because the other types of genetic elements are of lesser importance, far from actually, but mainly because the approaches to characterize them are either fairly straightforward (eg. INFERNAL ^60^ and tRNAscan-se ^61^ for non-coding RNA detection) or are the focus of more specialized analyses (eg. transcription factor binding sites).

The process of correctly determining the location and structure of the protein coding genes in a genome, “gene prediction”, is fairly well understood with many successful algorithms being developed over the past decades. In general, there are three main approaches to predict genes in a genome: intrinsic (or *ab-initio*), extrinsic and the combiners. Where the intrinsic approach focuses solely on information that can be extracted from the genomic sequence itself such as coding potential and splice site prediction, the extrinsic way uses similarity to other sequence types (e.g. transcripts and/or polypeptides) as information. There are inherent advantages and disadvantages to each of those.

The intrinsic approach is labor intensive as statistical models need to be built and software needs to be trained and optimized. Of prime importance for this approach is a good training set, i.e. a set of structurally well annotated genes used to build models and to train gene prediction software. As each genome is different, these models and software must be specific to each genome and thus need to be rebuilt and retrained for each new species. This is, however, also the big advantage of this approach, as it is capable of predicting fast evolving and species specific genes.

The extrinsic way, on the other hand, is much more universally applicable. A vast number of polypeptide sequences are already described and available in databases (eg. NCBI non-redundant protein, RefSeq, UniProt), which creates a wealth of information to be exploited in the gene prediction process. Transcript information, be it Sanger sequenced ESTs, RNA-Seq or even long read sequenced transcripts, plays an even bigger role in this approach. High quality protein sequences of other species provide good indication on the presence and location of genes and can be very useful to accurately predict the correct gene structure. Indeed, as polypeptide sequences often are more conserved than the underlying nucleotide sequences, they can still be aligned even from distantly related species. Although they are very useful to determine the presence of gene loci, they do not always provide accurate information on the exact structure of a gene. Transcripts on the other hand provide very accurate information for the correct prediction of the genes’ structure but are much less comprehensive and to some extent are noisier. Transcript information will not be available for all genes and sometime introns can still be present due to incomplete mRNA processing. Nonetheless, accurate alignment of the extrinsic data is key here: transcripts need to be splice-aligned (taking the exon-intron structure of eukaryotic genes into account) and protein sequences need to be compared to the six translation-frames of the nucleotide sequences. Moreover, it is a matter of thresholds: too stringent and less conserved genes will be missed, while too lenient will result in less specific information and introduce more false positives. These thresholds will depend on your objectives. A recommendation is to use lenient parameters in order to minimize the number of false negatives, as it is more difficult to create a new gene than to change the status of a false positive to obsolete. Then according to different confidence scores (e.g. coding potential, GO Evidence Codes), you can filter the gene set in order to provide, for instance, a high confidence gene set to train *ab initio* software, or a high confidence gene set to submit to a suitable repository and keep the full set for manual curation.

**Figure 3.**
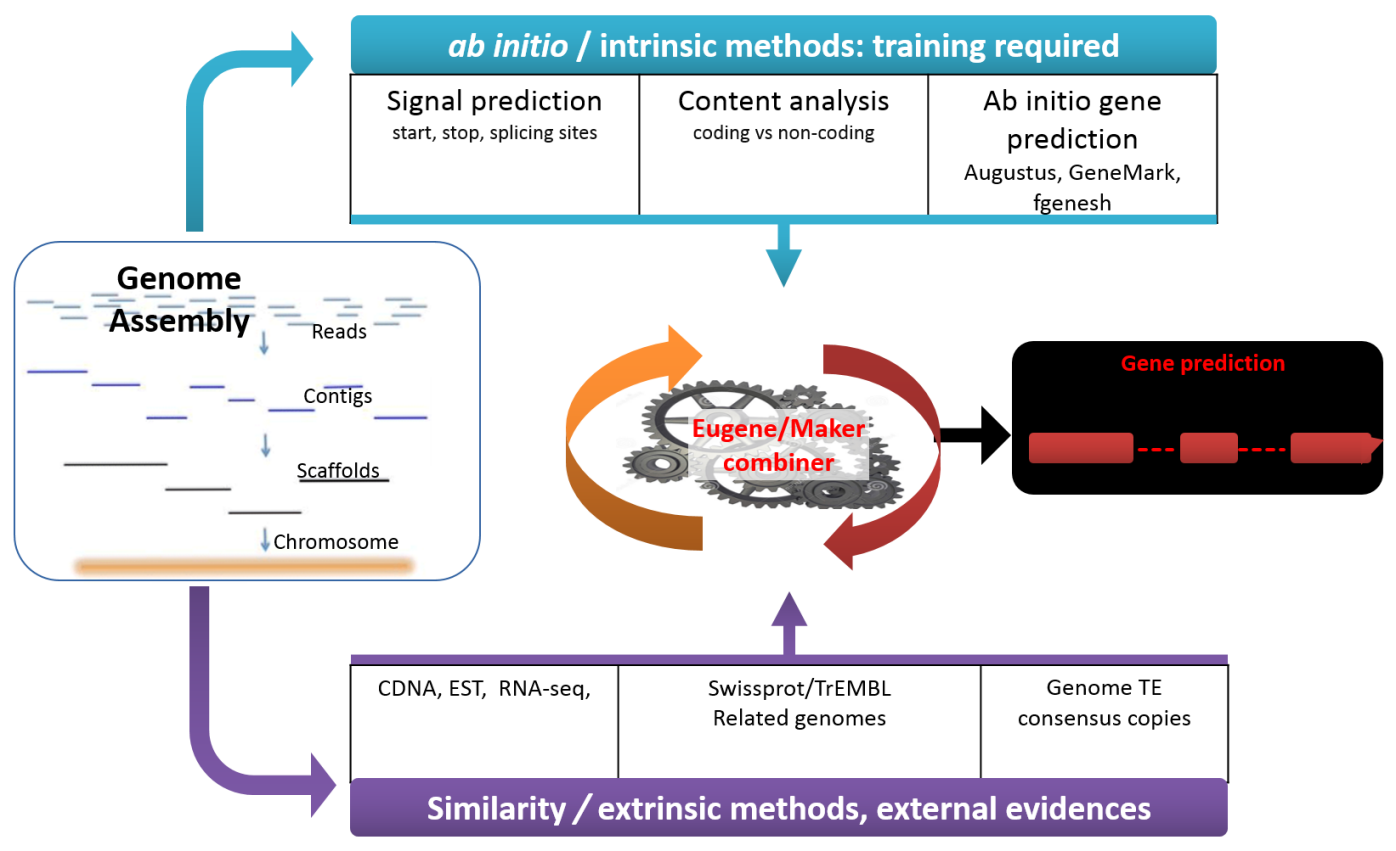
Simplified Illustration of a structural genome annotation using Combiners On the left, the diagram shows a typical assembly process. At the end of the process, scaffolds or chromosomes ready to be annotated are obtained. These scaffolds are then annotated using two different methods. The first method is called *ab-initio* and requires a known set of training genes. Once the *ab initio* tool has been trained it can be used to predict other similarly structured genes. The second similarity-based approach relies on experimental evidence such as CDSs, ESTs, or RNA-seq to build gene models. Combiners (such as Maker or Eugene) can then incorporate all of these results, eliminate incongruences, and present gene models best supported by all methods.

***The combiners are probably the most popular and widely used gene prediction approach.*** They integrate the best of both worlds: they have an *ab initio* part that is then often complemented with extrinsic information ( Figure 3). Especially, nowadays, with the advances of sequencing technologies, these approaches are increasingly used, reflecting the growing number of new tools and software trying to integrate RNA-Seq, protein or even intrinsic information. However, not all these combiners are the same. While some simply aim to pick the most appropriate model or build the consensus out of the provided input data (where an *ab initio* prediction tool might be one of them) for a given locus, others have a more integrated approach in which the intrinsic prediction can be modified by the given extrinsic data. The advantage of the latter is that they allow one type of information to overrule the other if this results in an overall more consistent prediction.

Apart from the choice of which tool to use, the choice of which data to integrate also has an influence on the final result. This is especially the case for the use of protein information. Error propagation is a real danger. Therefore, curated datasets, are preferred over the more general but less clean ones because it is vital that the provided information be as reliable as possible. The use of transcript information is less prone to error propagation although it is of importance that one realises what kind of data is being used. Short read RNA-Seq data is easily generated and is often an inherent part of a genome project. A downside is the short length of the reads. It will give accurate information on the location and existence of the exons but it will sometimes be more difficult to know how these exons are combined into a single gene structure. Therefore, it is becoming common to complement the short read transcript data with long read transcript information. Those will often contain the full set of exons into a single read and will as such provide unambiguous information on the complete gene structure and even alternative transcripts.

When performing genome annotation, choices have to be made, not only what tools to use but equally important what kind of data to use. It is clear that the choice should go towards the more reliable but unfortunately sometimes less comprehensive data sources as the use of lower quality information will inevitably lead to an inferior gene prediction result.

### 7.2. Functional annotation

**Figure 4.**
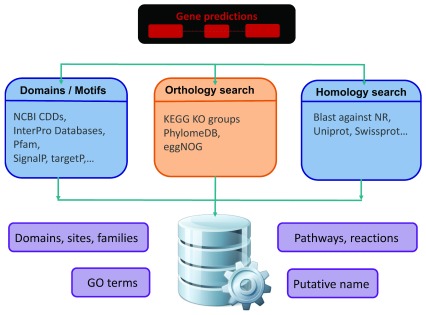
Functional Annotation Pipelines. This schema is showing a typical functional annotation pipeline, in which functional roles are assigned to coding sequences (CDSs) inferred in the gene prediction process. The process implements three parallel routes for the definition of functions. The first refers to proteins domains and motifs, the second for orthology search and finally the third is applied to homology search. At the end, the output from the three different sources is put together for more valuable predictions.

The ultimate goal of the functional annotation process ( Figure 4) is to assign biologically relevant information to predicted polypeptides, and to the features they derive from ( *e.g.* gene, mRNA). This process is especially relevant nowadays in the context of the NGS era due to the capacity of sequencing, assembling, and annotating full genomes in short periods of time, e.g. less than a month. Functional elements could range from putative name and/or symbols for protein-coding genes, e.g. ADH to its putative biological function, e.g. alcohol dehydrogenase, associated gene ontology terms, e.g. GO:0004022, functional sites, e.g. METAL 47 47 Zinc 1, and domains, e.g. IPR002328, among other features. The function of predicted proteins can be computationally inferred based on the similarity between the sequence of interest and other sequences in different public repositories, e.g. BLASTP against Uniprot. Caution should be taken when assigning results merely based on sequence similarity as two evolutionary independent sequences which share some common domains could be considered homologs ^62^. Thus, whenever possible, it is better to use orthologous sequences for annotation purposes rather than simply similar sequences ^63^. With the growing number of sequences in those public repositories, it is possible to perform various searches and combine obtained results into a consensus annotation. The accurate assignment of the functional elements is a complex process, and the best annotation will involve manual curation.

There are two main outcomes of the functional annotation process. The first is the assignment of functional elements to genes. Downstream analysis of these elements allow further understanding of specific genome properties, e.g. metabolic pathways, and similarities compared with closely related species. The second result of the functional annotation is the additional quality check for the predicted gene set. It is possible to identify problematic and/or suspicious genes by the presence of specific domains, suspicious orthology assignment and/or absence of other functional elements, e.g. functional completeness. These problematic genes can include those belonging to another species due to contamination, those detected as TEs, non-functional and/or artefactual genes annotated by error.

There are a number of tools available for functional annotation that allow users to obtain annotations for their gene set of interest via public databases in a high-throughput manner. These tools often start by sequence similarity search using tools like BLAST, HMMER or LAST against either non-redundant sequences database from NCBI GenBank and/or UniProt reference clusters (UniRef). After the initial homology search, candidate sequences can be assigned to one or more orthology groups using either best-reciprocal or tree-based methods ^63^. Alternatively, users can make use of machine learning methods, such as Hidden Markov Models (HMM) or neural networks to predict particular patterns from a given input gene set. The majority of these tools are freely available for the academic users, working under Linux OS and are often part of large-scale annotation pipelines.

For those users who do not want to run individual tools and combine results, there are a few available workflows that provide the entire annotation process. These pipelines can either include installation of the required tools and corresponding databases, or users are required to make this installation on their own and the pipeline just provides a framework for the analysis.

## 8. Use well-established output formats and submit your data to suitable repositories

### Data formats

The output of a genome annotation pipeline is almost always in GFF format. The information captured includes the structure and often the function of features of the genome, but usually not the actual sequence. Together with the Fasta file that was used in the annotation process, the sequence of these features can however easily be extracted. Other output formats are GTF, BED, Genbank, and EMBL, of which the last two include both sequence and annotation and are often used when submitting annotation results to sequence repositories. Some of these formats use controlled vocabularies and ontologies to guarantee interoperability between analysis and visualisation tools. We highly recommended the adoption of Fasta and GFF3 output formats. Both formats are compatible with the Genetic Model Organism Database (GMOD), a powerful suite of tools used for genome annotation, visualisation, and redistribution of genome data. By adhering to commonly used formats, you are making your results more useful to other researchers.

### Data submission

To improve the availability and findability of results from genome annotation projects, the annotated sequences have to be submitted to databases, such as Genbank at the National Center for Biotechnology Information (NCBI) ^64^ or the European Nucleotide Archive (ENA) ^65^. In these archives, the information relating to experimental workflows are captured and displayed. A typical workflow includes: 1) the isolation and preparation of material for sequencing, 2) a run of a sequencing machine in which sequencing data are produced, and 3) a subsequent bioinformatic analysis pipeline. ENA records this information in a data model that covers input information (sample, experimental setup, machine configuration), output machine data (sequence traces, reads and quality scores) and interpreted information (assembly, mapping, functional annotation).

There are also a growing number of theme-based genome databases. Human genome sequence projects are recommended to use the European Genome-phenome Archive (EGA) ^66^. EGA is a service for permanently archiving and sharing data resulting from biomedical research projects, and all types of personally identifiable genetic and phenotypic can be included. This service provides the necessary security to control access and maintain the confidentiality of patient data, while providing access to researchers and authorized physicians to view the data. The data was collected from individuals whose consent agreements authorize the disclosure of data only for specific investigations.

## 9. Ensure your methods are computationally repeatable and reproducible

Reproducibility and repeatability have been reported as a major scientific issue when it comes to large scale data analysis ^67^. For genomics to fulfil its complete scientific and social potential, *in silico* analysis must be both repeatable, reproducible and traceable. Repeatability refers to the re-computation of an existing result with the original data and the original software. For instance, the authors report numerical instability arising from a mere change of Linux platforms, even when using exactly the same version of the genomic analysis tools.

Fortunately, solutions exist and along with their report of numerical instability, the authors did show that repeatability could be achieved through the efficient combination of containers technology and workflow tools. Containers can be described as a new generation of lightweight virtual machines whose deployment has limited impact on performances. Container methods, such as Docker and Singularity, make it possible to compile and deploy a software in a given environment, and to later re-deploy that same software in the same original environment while being hosted on a different host environment. Once encapsulated this way, analysis pipelines were shown to become entirely repeatable across platforms.

Several workflow management systems, such as Nextflow, Toll and Galaxy, have recently been reported as having the capacity to use and deploy containers. These tools all share the same philosophy: they make it relatively easy to define and implement new pipelines, and they provide more or less extensive support for the massively parallel deployment of these pipelines across high performance computational (HPC) infrastructures or over the cloud.

Containerization also provides a very powerful way of distributing tools in production mode. This makes it an integral part of the ongoing effort to standardise genome analysis tools. The wide availability of public software repositories, such as GitHub or Docker Hub provides a context in which the implementation of existing standards bring immediate benefits to the analysis, both in terms of costs, repeatability and dissemination across a wide variety of environments.

The choice of a workflow manager and the proper integration of the selected pipelines through a well thought containerization strategy can therefore be considered an integral part of the genome annotation process, especially if one expects annotation to keep being updated over time. This makes the adoption of good computational practices like the one described here an essential milestone for genomic analysis to become compliant with the new data paradigm. In order to carry this out, the first guidelines to make data “findable, accessible, interoperable and re-usable” (FAIR) ^68^ was published in 2016. Even if FAIR principles were originally focused on data, they are sufficiently general so these high level concepts can be applied to any Digital Object such as software or pipelines.

Repeatability is merely the most technical side of reproducibility. Reproducibility is a broader concept that encompasses any decision and bookkeeping procedure that could compromise the reproducibility of an established scientific result. For this reason the implementation of the FAIR principle also impacts higher level aspect of the genome annotation strategy and for a genomic project to be FAIR compliant, these good practices should be applied to both data, meta-data and software. This can be achieved as follows:

### Data and meta-data

***Findable:*** Globally unique and persistent identifiers for data and metadata. Identifiers should persist across release and make it possible to trace back older analysis and relate them to the current annotation. Deprecated annotation should remain traceable. Even when data is not any longer available, meta-data should remain and provide a description of the original data.

***Accessible:*** Proper registration of data and metadata in suitable public, or self-maintained repository. All data should be properly indexed and searchable and accessible by identifier using standardized protocols

***Interoperable:*** Data and meta-data must be deposited using the most commonly used format

***Reusable:*** Data and meta-data standards should insure that the data is sufficiently well characterized to be effectively reused in future analysis or to be challenged by novel evaluation methods. Licensing should be as little restrictive as possible.

### Software and pipelines

***Findable:*** Software and pipelines should be deposited in an open source registries along with proper technical descriptions allowing their rapid identification.

***Accessible:*** Software should be deposited in public repositories such as GitHub, Docker Hub, so as to be available. Attempts should be made at having the licensing as little restrictive as possible. ELIXIR has taken the challenge to provide a long-term sustainable infrastructure to host software containers. Thus, this is the desirable solution to ensure software accessibility.

***Interoperable:*** Software should use the most common format and should be adequately documented. It should come along with a proper versioning for both the software and the reference biological databases they operate upon. The software behavior should also be adequately described using the right metadata, thus allowing programmatic interaction with other resources.

***Reusable:*** Software should be distributed in open-source format so as to ensure possible long term maintenance by third parties. Software should be encapsulated within containers ensuring the permanent availability of production mode pipelines. Authors should be encouraged to develop their pipelines in commonly used workflow managers (Galaxy ^69^, Nextflow ^70^, Snakemake ^71^). Decisions should be taken on the basis of a compromise between the level of usage of the selected workflow and its support of the required features. It should also contain meta-data describing which parameters have been used with the software in order to guarantee data reproducibility.

## 10. Investigate, re-analyse, re-annotate

Successful genome annotation projects do not just end with the publication of a paper; they should produce sustainable resources to promote, extend and improve the genome annotation life cycle.

Some genome consortia choose to manually review and edit their annotation data sets via jamborees, for instance the BioInformatics Platform for Agroecosystem Arthropods. Although this process is time- and resource-intensive, it provides opportunities for community building, education and training. All these elements help to improve the annotation life cycle and are promoted by the International Society for Biocuration.

Manual and continuous annotation are critical to achieve accurate and reliable gene models, mRNA, TEs, regulatory sequences, among other elements. In addition, research communities will face the generation of a huge volume of new data including re-sequencing, transcriptomics, transcriptional regulation profiling, epigenetic studies, high-throughput genotyping and other related whole-genome functional studies. Thus, it is important to provide a software infrastructure to facilitate the updating of the genomic data.

Tools such as WebApollo ^72^ from the GMOD project or web-portals like ORCAE ^73^ are particularly useful. These tools allow groups of researchers to review, add and delete annotations in a collaborative approach. The applications are robust and flexible enough to allow the members of a group to work simultaneously or at different times. The administration of the server allows to initiate a session to a user and if it has the authorization, to edit the content.

Thanks to this system, annotations of genomes can be improved in a continuous cycle as data is collected and updated. In this way the annotations can always continue to improve.

Other useful tools include Artemis ^74^, a successful curation software from the European Sanger institute and Gencode ^75^, which seems to succeed the Havana team’s Vega ^76^.

## Concluding remarks/general recommendations

Genome assembly and genome annotation are areas where there are no gold standards. Projects are often explorative, and knowing if your results are good or bad is often hard to determine. This is especially true if you are working with organisms only distantly related to already sequenced ones, which leaves you with little to compare with. Try to set an aim with your study, and stop working with the assembly and annotation once you have a result that allows you to reach that aim. Do not fall into the trap of wanting a “perfect” genome, as this tends to lead to a project that never ends. But also do not be afraid to start your own assembly and annotation project. With the development of new sequencing technologies it is more feasible than ever, and a well assembled and annotated genome will be a resource you can use for many years to follow.

The recommendations we give are broad guidelines, and we try not to force readers into explicit technologies or software. We do, however, present advantages of certain NGS technologies in specific cases, for example when looking at genome properties such genome size, complexity, or GC content. We also explain pitfalls to avoid throughout the whole assembly and annotation process. Finally, we also encourage the adoption of our guidelines regarding data deposition and reproducibility, as they offer a simple mechanism to improve the quality, findability, reusability and sustainability of results derived from genome assembly and genome annotation projects.
