# Supplementary material for: Ten steps to get started in Genome Assembly and Annotation
Source: F1000Res. 2018 Feb 5;7:ELIXIR-148. [Version 1] doi: 10.12688/f1000research.13598.1 (PMC5850084; doi:10.12688/f1000research.13598.1)
Supplement: Peer review history

# Peer review: Referee response for version 1

**DOI:** 10.5256/f1000research.14771.r30566

**Published:** 2018-03-12

**Authors:** Dave Clements

**Affiliations:** 1 Department of Biology, Johns Hopkins University, Baltimore, MD, USA

This paper does a good job of covering the big picture of what's needed to assemble and annotate a genome.  Its stated goal is to give guidelines that are "broadly applicable" and "intended to be stable over time."   This paper achieves that goal, and all of my concerns are minor.  However, "stable over time" was a frustrating goal for me.  This means that comments on specific sequencing technologies and software are not as common as they would be in a review article on the state of the art.  This is a fine line to walk.

 Specific comments

**Introduction**

 1. Think about dropping or shortening the checklist at the end of the introduction.  I believe that all of this is covered in detail in the individual sections.

**Repeats**

 1. "Contigs" used for the first time,  Not sure if these need to be explained.  (Could reference figure 3.)

**Chemical purity**

 1. What ONT means is explained later.  Move that explanation here.

 2. The text says:

   It is widely known in the PacBio community that samples rich in contaminants...

 Are there any references for this?  This highlights a larger question with the paper.  It is an *opinion* article and it contains many opinions such as 

   The characteristics of the genomes being assembled have a greater impact on the results than the choice of the algorithm.

 I am not disputing any of these statements, but if and when references exist that support them, then those references should be included.

**Long read genome assembly**

 This section says error rates are 5-20%.  Elsewhere in the paper they are given as "up to 10% or 15%."

**Scaffolding and gap filling**

 The very end of this section states:

   Be aware that any changes to a genome assembly will most likely necessitate annotation to be re-started from scratch, and you should therefore be sure to “freeze” the assembly completely before starting annotation.

 I think "restarted from scratch" gives the wrong impression.  Tools such as MAKER can do liftover from one version of an assembly to the next.  Perhaps this could be clarified?

**Ensure your methods are computationally repeatable and reproducible**

 Toll -> Toil ?

I have read this submission. I believe that I have an appropriate level of expertise to confirm that it is of an acceptable scientific standard.

# Peer review: Referee response for version 1

**DOI:** 10.5256/f1000research.14771.r31487

**Published:** 2018-03-09

**Authors:** Bruno Contreras-Moreira

**Affiliations:** 1 Estación Experimental de Aula Dei-CSIC, Fundación ARAID, Zaragoza, Spain

The opinion article by Victoria Dominguez Del Angel and collaborators is a well-written sort of broad tutorial which will certainly be of help for anyone trying to sequence and assemble a genome sequence with little previous experience. While it avoids details that are required when the real work is actually carried out (for instance, K-mer length), it discusses important questions that must be addressed before carrying out genomic projects, and choices that must be made along the way down to the point that data and procedures are published and released.

 Next I comment on specific parts of the text that I believe can be improved.

**0. Introduction**

 0.1 Genomics in 2018 cannot possibly be done without a pan-genome perspective. This has been true for years in the area of microbiology, where projects now rarely assemble and annotate a single genome, but rather a few dozens related strains. In addition, this is becoming state-of-the-art also for genomic projects of crops and model plants, such as *Brachypodium distachyon*, as well as in human medicine. A couple of sentences should be added explaining that in this context a group of genomes are sequences, assembled and annotated in parallel, which makes it more challenging but also facilitates spotting and correcting errors.

 0.2 I would add to the checklist a literature survey to identify related genomes.

**1. Investigate the properties of the genomes you study**

 1.1 Heterozygosity

 I would add a sentence about the outcrossing or selfing nature of species, which has a direct impact on the expected heterozygosity, and often limits the possibility to obtain inbred individuals. In plants double-haploids are used to this end (see for instance ^1^.

 1.2 Ploidy level

 Please note that it has been estimated that many plant species are polyploid ^2^^-^^3^. One strategy to solve these complex genomes is to first sequence the genomes of the expected/known parental species.

**2. Extract high quality DNA**

 2.1 Organelle DNA

 I would add that frequently chloroplast genomes or plastomes are of high interest as they can provide a complementary, maternally-biased evolutionary history. This might be of particular interest for polyploid species as it might help determine which was the maternal and which the paternal genome donor. Even with a low ratio of organelle DNA in our experience is likely that complete chloroplasts can be assembled and annotated (see for instance ^4^) as a by-product of whole genome sequencing. Instead, mitochondria seem to be difficult to assemble in plants.

**4. Estimate the necessary computational resources**

 I would add that the assembly tools selected at the time the proposal was written are likely to be replaced by others when the work is actually to be performed due to pace of innovation in this area.

**5. Assemble your genome**

 In the last left-side paragraph it is said that “misassemblies in an existing assembly need to be broken prior to scaffolding in order to join the correct contigs together.” . This is followed by another sentence later on “Unfortunately, there are few ways to distinguish what is real, what is missing, and what is an experimental artefact.”

 In our experience many scaffolding errors can be spotted by mapping back the original sequence reads to the assembly and then visualizing the results with browsers such as IGV. Of course this can be cumbersome for very large assemblies, but tools such as SEQuel and Reapr can be really helpful for such tasks.

 5.1 BUSCO is shown as a way of evaluating assembly quality in page 10.

 I would add that in pan-genome projects this can be generalized so that assemblies, and subsequent annotations, can be evaluated in terms of the proportion of core-genes contained. Poor assemblies can be identified frequently for having less core-genes than expected.

**7. Annotate genes with high quality experimental evidence**

 I feel this section can be improved by:

 7.1 Stressing that different annotation strategies often yield annotation sets that are implicitly biased. Therefore, if the user plans to compare its genome to others should make and effort to use a similar approach so that any conclusions regarding issues such as gene family expansions are not caused by the underlying methodology. Indeed we have seen this happening when annotating a microbial pan-genome and then comparing it to genomes in public databases.

 7.2 Adding a section on microbial genome annotation, mentioning popular tools such as PROKKA, RAST or NCBI Prokaryotic Genome Annotation Pipeline, and commenting on the annotation of bacterial features such as CRISPRs or plasmids.

 7.3 On page 12, 1 ^st^ paragraph, is its stated that “Transcripts on the other hand provide very accurate information for the correct prediction of the genes”

 I think it should definitely be mentioned that, unlike HQ protein sequences, transcripts allow the annotation of unstranslated regions (UTR) and despite their noise and the isoform deluge can be used to define also gene promoters, which can then be annotated in terms of regulation.

 7.4 I miss a section on comparison of gene order/synteny

**8. Use well-established output formats and submit your data to suitable repositories**

 I would add that soft/hard repeat-masked versions of the genome sequence can be made available in FASTA format, which are helpful for subsequent analyses of regulatory sequences.

**Minor edits:**

 Page 3, 1 ^st^ paragraph:

 “The advice here presented is based on a need seen while working in the ELIXIR-EXCELERATE task “Capacity Building in Genome Assembly and Annotation”. In this capacity we have”

 can be changed to

 “The advice here presented is based on a need first seen while working in the ELIXIR-EXCELERATE task “Capacity Building in Genome Assembly and Annotation”. In this project we have”

 Page 6, 2 ^nd^ paragraph

 Why is “or after” in bold?

 Page 14, left column

 Fasta format should be FASTA format

I have read this submission. I believe that I have an appropriate level of expertise to confirm that it is of an acceptable scientific standard.

## References

[1] DaccordNCeltonJMLinsmithGBeckerCChoisneNSchijlenEvan de GeestHBiancoLMichelettiDVelascoRDi PierroEAGouzyJReesDJGGuérifPMurantyHDurelCELaurensFLespinasseYGaillardSAubourgSQuesnevilleHWeigelDvan de WegETroggioMBucherE: High-quality de novo assembly of the apple genome and methylome dynamics of early fruit development. *Nat Genet*.2017;49(7) : 10.1038/ng.3886 1099-1106 10.1038/ng.3886 28581499

[2] OttoSPWhittonJ: Polyploid incidence and evolution. *Annu Rev Genet*.2000;34: 10.1146/annurev.genet.34.1.401 401-437 10.1146/annurev.genet.34.1.401 11092833

[3] DoyleJSherman-BroylesS: Double trouble: taxonomy and definitions of polyploidy. *New Phytologist*.2017;213(2) : 10.1111/nph.14276 487-493 10.1111/nph.14276 28000935

[4] SanchoRCantalapiedraCPLópez-AlvarezDGordonSPVogelJPCatalánPContreras-MoreiraB: Comparative plastome genomics and phylogenomics of Brachypodium: flowering time signatures, introgression and recombination in recently diverged ecotypes. *New Phytol*.2017; 10.1111/nph.14926 10.1111/nph.14926 29206296
